# Diabetes mellitus and intermittent claudication: a cross-sectional study of 920 claudicants

**DOI:** 10.1186/1758-5996-6-21

**Published:** 2014-02-17

**Authors:** Francisco S Lozano, José R González-Porras, José R March, José M Lobos, Eduardo Carrasco, Eduardo Ros

**Affiliations:** 1 Hospital Universitario de Salamanca e IBSAL, Salamanca, Spain; 2 Hospital Universitario de Getafe, Madrid, Spain; 3 Centro de Salud Villablanca, Madrid, Spain; 4 Centro de Salud Jesús H. Gómez Tornero, Abarán, Murcia, Spain; 5 Hospital Universitario San Cecilio, Granada, Spain; 6 Servicio de Angiología y Cirugía Vascular, Hospital Universitario de Salamanca, Universidad de Salamanca, Paseo de San Vicente s/n, Salamanca, 37007, Spain

**Keywords:** Diabetes mellitus, Intermittent claudication, Ankle-Brachial index, Walking impairment questionnaire, Quality of life, EuroQol

## Abstract

**Introduction:**

Diabetes mellitus (DM) and intermittent claudication (IC) are frequently associated health conditions. Our hypothesis is that the nature, severity and quality of life (QoL) of patients with IC and DM are worse than those of claudicant patients without diabetes.

**Material and methods:**

An observational, cross-sectional and multicentre study of 920 patients with IC, divided into two groups: diabetic (n = 477) and non-diabetic (n = 443). For each group, we examined clinical and biological characteristics (including levels of glucose and lipids), the ankle-brachial index (ABI), responses to the Walking Impairment Questionnaire (WIQ) and the European Quality of Life-5 Dimensions (EQ-5D) questionnaire.

**Results:**

Compared with claudicant patients without diabetes, claudicants with diabetes were older (p < 0.001), more likely to be female (p = 0.006), with a higher BMI (p < 0.001), more likely to have a sedentary lifestyle (p < 0.001) and to be a non-smoker (p < 0.001). Claudicant patients with diabetes also had significantly more cardiovascular risk factors (p < 0.001), more frequent ischaemic cardiopathy (p = 0.023) and chronic renal failure (p = 0.002), and fewer prior ictus events (p = 0.003). No significant differences between groups were found with respect to blood pressure, levels of cholesterol or triglycerides. The mean ABI of diabetic-IC patients was slightly lower than IC patients without diabetes (p = 0.016). All WIQ subdomains scores were significantly lower (p < 0.001), indicating poorer walking ability, in claudicant and diabetic patients with compared with those without diabetes. The mean E5-QD global scores and the mean EQ-5D visual analogue scale in the whole series were 0.58 (SD = 0.21) and 55.04 (SD = 21.30), respectively. Both E5-QD scores were significantly lower, indicating poorer QoL, in claudicant patients with diabetes than claudicant patients without diabetes (p < 0.001).

**Conclusion:**

Patients with IC and DM had more risk cardiovascular factors, cardiovascular conditions, disability and worse haemodynamic status and QoL than claudicant patients without diabetes.

## Introduction

Peripheral arterial disease (PAD) of the legs has a range of different clinical presentations, from pain with walking (intermittent claudication; IC) to gangrene. IC is always accompanied by different levels of incapacity and morbidity-mortality (cardiovascular disease-associated) that significantly impair patients’ quality of life (QoL) [1]. The prevalence of IC is particularly high and variable, as illustrated by the studies reviewed herein. In the TASC-I study the prevalence of IC ranged from 0.4% to 14.4% [2]. A cross-sectional study of 1324 participants aged 55 to 84 years conducted in Spain found a prevalence of IC of 8.03% [3].

Diabetes mellitus (DM) is considered the “epidemic of the 21^st^ century”. The prevalence of DM for all age groups worldwide was estimated to be 2.8% (171 million) in 2000, a figure that was predicted to double over the subsequent two decades [4]. DM is a well-established risk factor for arteriosclerosis obliterans and diabetic patients often have IC [5]. According to the Framingham study, there has been a steady rise in the prevalence of diabetes among claudicant patients, from 2% during 1950 – 1969 to 25% during 1990 – 1999 [6].

Despite these dramatic figures, there is surprisingly little information about diabetic claudicants, especially if we compare this with other phases (critical limb ischaemia) or the process of PAD (diabetic foot) (Table 1). In the line of Dolan et al. [7] it is important to recognize the presence of IC in DM patients to prevent most advances stages of the PAD, frequently lead to the loss of the limb. This prompted us to test the hypothesis that the nature, severity and QoL of patients with IC and DM are worse than those of claudicant patients without diabetes.

**Table 1:** Distribution of bibliographic references indicating the fewer studies on intermittent claudication in diabetes mellitus patients, derived from a literature search in PubMed (up to 3 January 2013)

**Keyword**	**Number of references**	**References in last 5 years**
Diabetes Mellitus (DM)	322,586	76,595 (23.7%)
Peripheral Arterial Disease (PAD)	59,401	8,167 (13.7%)
Intermittent Claudication (IC)	7,999	1,147 (14.3%)
PAD & DM	3,777	1,067 (28.3%)
IC & DM	667	103 (15.4%)
Diabetic Foot (DF)	8,794	2,597 (29.5%)

Note: The IC & DM group contains 0.2% of the articles about diabetes, 1.7% of those about PAD and 8.3% of those about IC. There is one article about IC & DM for every 10 articles about DF.

## Material and methods

An observational, prospective, cross-sectional and multicentre study (covering the entire country) was undertaken between May and December 2011. The study was approved by the Scientific and Ethics Committee of the Clinical Hospital, Barcelona, Spain. The study was carried out by vascular surgeons, members of the Spanish Society for Angiology and Vascular Surgery. Each participating investigator included 3–5 consecutive patients with IC (with or without associated DM).

### Study population

1,192 patients with IC at least one year before the start of study were recruited from the entire Spanish territory by 356 vascular surgeons (VS). VS participants were invited, via a letter, by an *ad hoc* committee. All VS participants are active members of the Spanish Society of Vascular Angiology and Surgery and were selected according to predefined selection criteria: a) involvement in our Society, and b) even geographical distribution. None of the VS invited refused to participate in the study.

The IC diagnosis was ensured by medical history (including Edinburgh questionnaire), physical exploration and Ankle-brachial Index [3]. All included patients gave their informed consent to enter the study. Criteria for inclusion from the study were: a) lower limb intermittent claudication (Fontaine grade II), lasting at least 1 year; b) man or woman; c) aged between 45-85 years; e) any race. Criteria for exclusion from the study were: a) patient did not give informed consent to take part in the study; b) patient with recent admission or with a terminal illness; c) presence of systemic diseases that could cause an overall total reduction in mobility (e.g., congestive heart disease with oxygen therapy, rheumatic diseases and wheelchair, etc.); d) patient had undergone surgery within the past 6 months due to limb peripheral artery disease; e) patient had a major psychiatric condition; f) alcohol or drug addiction; g) critical arterial ischaemic limb; h) patient unable to answer the questions in the WIQ and/or EQ-5D questionnaires; i) patient unable to understand the instructions of the study. Patients had to meet all inclusion criteria in order to be eligible. Some who were otherwise suitable for inclusion were withdrawn from the study because they had incomplete clinical questionnaires (< 80% of items), their ankle-brachial index had not been recorded, or the WIQ or EQ-5D questionnaire had not been fully completed.

### Measurement instruments

For each patient, the investigator completed a Clinical Questionnaire (CQ) covering three areas: 1) demographics and clinical data; 2) blood pressure and laboratory tests (glycaemia and lipids levels); and 3) ankle-brachial index (ABI). Additionally, each patient included in the study filled in two questionnaires: the Walking Impairment Questionnaire (WIQ) and the European Quality of Life-5 Dimensions (EQ-5D) questionnaire.

#### Ankle-brachial Index (ABI)

A portable Doppler apparatus was used (8 MHz probe). Techniques were applied according to the recommendations of the American Heart Association [8]: patient supine and at rest. For each patient, systolic blood pressures were measured in the following order: dorsalis pedis and posterior tibial arteries of each leg and both brachials. The ABI of each extremity was calculated by dividing the highest pressure obtained in either of the leg arteries by the maximum brachial value. In the records of each patient only the claudicant limb, or the lower ABI in the bilateral cases, was taken into account. An ABI between 0.90 and 1.2 was considered normal (no patients included). Values less than 0.90 were consistent with PAD. An ABI value greater than 1.3 was also considered abnormal, suggesting calcification of the arterial walls; such values are often found in diabetic patients.

#### Walking Impairment Questionnaire (WIQ)

WIQ is a brief, easy-to-complete, disease-specific questionnaire specifically designed for assessing the consequences of IC [9]. WIQ has been validated against treadmill walking test and provides a subjective patient report of functional walking ability. WIQ is not a QoL questionnaire but may be used alone or in conjunction with QoL questionnaires and/or a treadmill exercise test. The questionnaire addresses aspects related to walking distance, walking speed, and stair-climbing capacity during the previous month. For walking distance, the questionnaire considers the degree of difficulty in walking specified distances. The degree of difficulty for the corresponding task is expressed on an ordinal scale from 0 (complete difficulty) to 4 (full capacity to perform the task). Each domain is represented as a score [(Individual Score/4) × 100] ranging from 0% (complete incapacity) to 100% (full capacity). The questionnaire typically takes around 5 minutes to complete and has been validated for interviewer administration or self-administration. We used the validated Spanish version [10].

#### European Quality of Life-5 Dimensions (EQ-5D) questionnaire

EQ-5D was designed by a European group [11]. It is a QoL general questionnaire widely employed in research because of its ease of use. It consists of three parts. The first captures health-related quality of life across five domains: mobility, self-care, usual activities, pain/discomfort, and anxiety/depression. The scores obtained are summarised as an overall index between 0 (worst health status) and 1 (best health status). The second part consists of a 20-cm, vertical, visual analogue scale (VAS), in the form of a thermometer, with endpoints of worst and best imaginable health status (scored 0 and 100, respectively). The third part (the EQ-5D Index) measures a series of societal preference values and was not evaluated in this study. The EQ-5D has been tested and validated in patients with IC [12]. We used a Spanish version [13].

### Statistical analysis

All data were anonymously documented to ensure confidentiality, and collated in a PASW version 18 (IBM, New York, USA). Continuous variables were summarised as the mean and standard deviation (SD); discrete variables were expressed as percentages. Continuous variables with a Gaussian distribution were compared using unpaired t-tests, while those with a non-Gaussian distribution were compared using the Mann–Whitney test. Discrete variables were compared by the χ^2^ or Fisher´s exact test. Fisher’s exact test was performed when expected percentages were less than 5.

## Results

**Figure 1:**
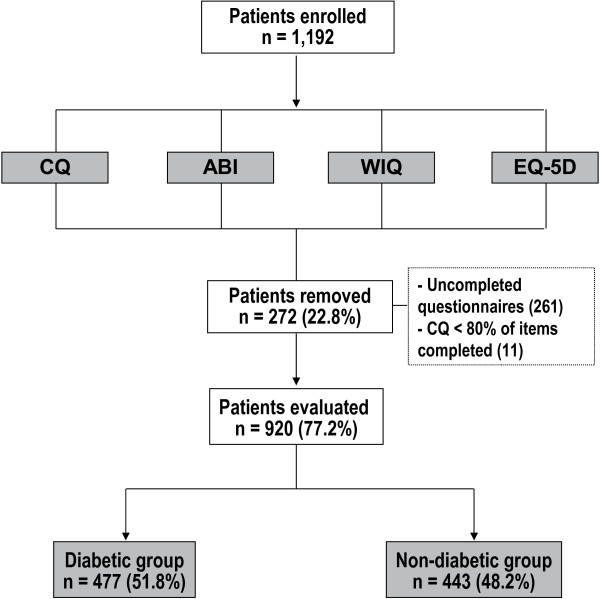
**Study design.** CQ = Clinical Questionnaire; ABI = Ankle-Brachial Index; WIQ = Walking Impairment Questionnaire; EQ-5D = European Quality of Life-5 Dimensions; DM = Diabetes Mellitus.

Of the 1192 patients with IC included in the study, 272 were removed (148 and 124 in the diabetic and non-diabetic groups respectively) because their data were incomplete (mainly due to the failure to total complete the WIQ or EQ-5D questionnaire). The sample was divided into two groups: with and without diabetes mellitus (n = 477, 51.8% and n = 443, 48.2%, respectively) (Figure 1).

### Clinical features of patients by diabetes status

The main clinical features of the patients with IC with respect to the presence or absence of diabetes are listed in Table 2. Overall, there was a clear predominance of men (77.9%), sedentary lifestyles (57.4%) and a family history of cardiovascular disease (58.8%). The mean age of the total series was 68.2 (SD = 9.8) years and the mean BMI was 27.6 (3.6). Compared with claudicant patients without diabetes, claudicants with diabetes were older (p < 0.001), more likely to be female (p = 0.006), with a higher BMI (p < 0.001) and more likely to have a sedentary lifestyle (p < 0.001). Claudicant patients with diabetes more often had hypertension (p < 0.001) than those without diabetes. Almost 48.2% of claudicants with diabetes had at least three risk factors for cardiovascular disease, compared with only 16.3% of claudicants without diabetes. On the other hand, active smoking was significantly more frequent in non-diabetic than in diabetic claudicants (p < 0.001).

**Table 2:** Characteristics of the groups and overall series

**Characteristic**	**Diabetic (n = 477)**	**Non-diabetic (n = 443)**	**Total (n = 920)**	p*
Male (n, %)	355 (74.4)	362 (81.7)	717 (77.9)	0.006
Year (mean, SD)	69.8 (9.3)	66.4 (10.0)	68.2 (9.8)	< 0.001
BMI (mean, SD)	28.2 (3.7)	26.9 (3.4)	27.6 (3.6)	< 0.001
Sedentarism (n, %)	317 (66.5)	211 (47.6)	528 (57.4)	< 0.001
Ex-smoker (n, %)	178 (37.3)	171 (38.6)	349 (37.9)	0.085
**Cardiovascular (CV) risk factor (n, %)**				
Smoker	168 (35.2)	189 (42.7)	357 (38.8)	< 0.001
Diabetic mellitus	477 (100)	0 (0)	477 (100)	< 0.001
Hypertension	385 (80.7)	313 (70.7)	698 (75.9)	< 0.001
Dyslipidaemia	311 (65.2)	280 (63.2)	591 (64.2)	0.528
Number of CV risk factors				
None	0 (0.0)	23 (5.2)	23 (2.5)	< 0.001
One	29 (6.1)	130 (29.3)	159 (17.3)	
Two	125 (26.2)	218 (49.2)	343 (37.3)	
Three	230 (48.2)	72 (16.3)	302 (32.8)	
All (four)	93 (19.5)	0 (0.0)	93 (10.1)	
**CV disease-associated conditions (n, %)**				
Cardiac failure	50 (10.5)	36 (8.1)	86 (9.3)	0.220
Ischaemic cardiopathy	160 (33.5)	118 (26.6)	278 (30.2)	0.023
Arrythmias	54 (11.3)	31 (7.0)	85 (9.2)	0.024
Valvulopathy	22 (4.6)	11 (2.5)	33 (3.6)	0.083
Chronic renal failure	53 (11.1)	24 (5.4)	77 (8.4)	0.002
**Prior CV diseases (n, %)**				
Myocardial infarction	62 (14.0)	67 (14.0)	129 (14.0)	0.982
Angina pectoris	38 (8.6)	48 (10.1)	86 (9.3)	0.439
Stroke	4 (0.9)	19 (4.0)	23 (2.5)	0.003
**Osteoarticular diseases (n, %)**				
Arthrosis	263 (55.1)	158 (35.7)	421 (45.8)	< 0.001
Arthritis	21 (4.4)	21 (4.7)	42 (4.6)	0.806
Vertebral disc prolapse	45 (9.4)	36 (8.1)	81 (8.8)	0.484
Lumbar pathology	83 (17.4)	69 (15.6)	152 (16.5)	0.456
Major trauma	5 (1.0)	4 (0.9)	9 (1.0)	1.000
Family history of				
CV disease (n, %)	295 (61.8)	246 (55.5)	541 (58.8)	0.095

BMI = Body mass index.

*Diabetic group vs non-diabetic group.

The most common cardiovascular disease associated with IC was ischaemic cardiopathy (30.2% of the total series). Compared with non-diabetic claudicants, those with diabetes more frequently exhibited ischaemic cardiopathy (33.5% *vs*. 26.6%, p = 0.023) and chronic renal failure (11.1% *vs*. 5.4%, p = 0.002). Other cardiovascular diseases (congestive cardiac failure, arrhythmias or valvulopathies) were similarly less frequent in both groups.

There were histories of myocardial infarction or angina pectoris of 14.0% and 9.3%, respectively, in the whole series. No significant differences between claudicant patients with or without diabetes were found with regard to the history of myocardial infarction or angina pectoris, but a history of stroke was more frequent in claudicant patients without diabetes than in those with diabetes (p = 0.003).

The most common osteoarticular comorbid conditions were arthrosis (45.8%) and lumbar pathology (16.5%). Arthrosis was significantly more frequent in IC with than without diabetes (55.1% *vs*. 35.7%, p < 0.001). Lumbar pathology and other osteoarticular comorbidities (arthritis, vertebral disc prolapse and major trauma) occurred at similarly lower frequencies in the two groups.

No significant differences between the groups were found with regard to blood pressure, levels of cholesterol and triglycerides. The mean ABI of the total series was 0.63 (SD = 0.19). The ABI was significantly lower in claudicant patients with than without diabetes (0.62 vs. 0.64, p = 0.016). An ABI less than 0.50 was detected in 170 claudicant patients, such values being more frequent amongst those with diabetes than those without (22.0% *vs*. 14.7%, p = 0.033).

### WIQ and EQ-5D of patients by diabetes status

WIQ and EQ-5D scores are shown in Table 3. The mean walking distance, walking speed and stair-climbing capacity in the entire series were 34.07 (SD = 26.77), 35.43 (SD = 23.0) and 41.16 (SD = 28.54), respectively. All WIQ subdomains scores were significantly lower, indicating poorer walking capability, in claudicant patients with diabetes than in claudicant patients without diabetes (p < 0.001 for all comparisons). Pain was more severe in claudicants with diabetes than in those without (44.29 *vs*. 50.17, p < 0.001).

**Table 3:** Summary values for characteristics of groups and the overall series

**Characteristic**	**Diabetic (n = 477)**	**Non-diabetic (n = 443)**	**Total (n = 920)**	p*
**SBP (mm Hg)**	144.0 (17.8)	142.8 (17.5)	143.4 (17.7)	0.540
**DBP (mm Hg)**	81.6 (10.5)	81.5 (10.6)	81.5 (10.6)	0.647
**Analytical (mg/dl)**				
Glycaemia	155.2 (45.2)	101.2 (21.6)	130.1 (45.1)	< 0.001
Cholesterol	207.3 (49.2)	209.8 (44.8)	208.4 (47.1)	0.244
HDL	51.7 (28.8)	52.1 (26.6)	51.9 (27.8)	0.078
LDL	125.9 (40.2)	130.8 (39.1)	128.2 (39.7)	0.118
Triglycerides	161.6 (75.4)	155.7 (79.7)	158.9 (77.4)	0.102
**ABI**				
Overall	0.62 (0.21)	0.64 (0.15)	0.63 (0.19)	0.016
Categories (n,%)				
> 1.30	29 (6.1)	26 (5.9)	55 (6.0)	0.033
0.90-0.50	343 (71.9)	352 (79.4)	695 (75.5)	
< 0.50	105 (22.0)	65 (14.7)	170 (18.5)	
**WIQ**				
Pain (mean%)	44.29 (20.05)	50.17 (20.35)	47.12 (20.40)	< 0.001
Walking distance	1395.60 (1136.22)	1811.26 (1337.63)	1595.75 (1253.96)	< 0.001
Distance (mean%)	29.79 (24.26)	38.67 (28.56)	34.07 (26.77)	< 0.001
Walking speed	14.54 (9.65)	18.19 (11.21)	16.30 (10.59)	< 0.001
Speed (mean%)	31.62 (20.99)	39.54 (24.38)	35.43 (23.00)	< 0.001
Stair-climbing	106.11 (76.48)	131.92 (86.01)	118.54 (82.19)	< 0.001
Stair-climbing (mean%)	36.84 (26.56)	45.81 (29.86)	41.16 (28.54)	< 0.001
**EQ-5D**^**†**^				
Global score	0.54 (0.22)	0.62 (0.19)	0.58 (0.21)	< 0.001
VAS score (mean%)	53.31 (20.54)	56.92 (21.97)	55.04 (21.30)	0.003

^†^0-1 or 0-100% (worst – best quality of life).

SBP = Systolic blood pressure.

DBP = Diastolic blood pressure.

HDL = High-density lipoprotein: LDL = Low-density lipoprotein.

ABI = Ankle-brachial index; WIQ = Walking Impairment Questionnaire; EQ-5D = EuroQol.

*Diabetic group vs non-diabetic group.

Values are the mean (and standard deviation), except where indicated as being frequency and percentage.

The mean E5-QD global score and the mean EQ-5D visual analogue scale in the overall series were 0.58 (SD = 0.21) and 55.04 (SD = 21.30), respectively. Both E5-QD scores were significantly lower, indicating poorer QoL, in claudicant patients with diabetes than in those without diabetes (p < 0.001).

## Discussion

Our results indicate that claudicant patients with diabetes have some distinct clinical characteristics as well as more severe disease and poorer QoL than claudicant patients without diabetes.

The medical-social and economic consequences of PAD and IC are very important, as is apparent from the extensive bibliography. PAD is a major public health problem in western countries. IC is a common, chronic disease that can cause injuries throughout a substantial portion of a patient’s life. The true prevalence of PAD in people with diabetes has been difficult to determine because most patients are asymptomatic. Population-based studies have revealed a prevalence of PAD in people with diabetes of up to 30% [14]. PAD is equally prevalent in type 1 and type 2 diabetes, although some authors report a higher prevalence in type 2 diabetes, which is the more common diabetes [5]. According to the HUNT study, the prevalence of IC in patients with DM was more than three times that in non-diabetic participants [15]. However, it is significant and notable that the prevalence of claudication in the diabetic population with PAD is lower than that of critical limb ischaemia (CLI) in this population [16]. The characteristics of the macro- and micro-angiopathy in the diabetic population lead to an IC that is less benign than that in the non-diabetic population, and the PAD in diabetic patients would be much more pronounced and the progression of the disease more rapid. Therefore, it is important to detect and measure the characteristics of IC in the diabetic population.

The characteristics of our series are quite similar to those of other series examined throughout the world [6,17]. Diabetic claudicants have a substantial burden of cardiovascular risk factors, prior cardiovascular disease or cardiovascular disease-associated conditions. However, we have not found any satisfactory explanation for the lower prevalence of smoking and history of stroke among diabetic PAD/IC patients. The association of osteoarticular disease in claudicant patients is a phenomenon that has been little studied. Breek et al. [18] showed that the back, hip and knee symptoms were very prevalent in IC. In our series, up to half of the claudicant patients had arthrosis, and their walking capability and QoL could be partially predicted by the presence of osteoarticular comoborbidity.

As other authors have observed [19,20], we found statistically significant differences in the level of lipids between diabetic and non-diabetic claudicants.

The mean ABI in our total series was 0.63 (SD = 0.19). Similar mean ABIs have been found in previous studies (ranging from 0.50 to 0.76; median = 0.62) [20-24]. The mean ABI for diabetic PAD patients was slightly lower than that for PAD without diabetes [7,20,25], this difference being statistically significant in our study.

However, the most outstanding feature of ABI in the diabetic population is its diagnostic and prognostic value [25]. ABI thresholds of less than 0.9 and more than 1.3 are highly suggestive of PAD in diabetic patients [26] while, conversely, diabetics with an ABI > 1.40 have a poorer prognosis [27].

Walking deterioration measured by the WIQ in our series was reasonably consistent with the results of previous studies [7,9,22,28]. The mean estimate of pain was 47.1 (range in previous studies = 39.6 – 63.3), walking distance was 34.1 (range in previous studies = 22.6 – 55.0), walking speed was 35.4 (range in previous studies = 23.9 – 48.3) and stair-climbing capacity was 41.2 (range in previous studies = 23.8 – 65.0). We should emphasise that walking deterioration was significantly poorer in claudicant patients with diabetes than in those without, as other authors have reported [7,19,20].

It is well know that PAD is associated with a significant reduction in quality of life. Specifically, IC represents a deterioration in QoL compared with controls [29]. Accordingly, the mean QoL measured by EQ-5D in our series (0.58, SD = 0.21) is similar to the values from studies from various parts of the world. Again, claudicant patients with diabetes had poorer QoL than their counterparts without diabetes [19,30-33], irrespective of the type of diabetes [18,30-32].

Atherosclerotic disease, specifically angina pectoris, causes a greater deterioration of QoL in IC [29]. As our study indicates, the presence of atherosclerotic disease is more frequent in diabetes. Osteoarticular disease can also affect walking [18]. It is possible that this comorbidity might partly explain the worse QoL of diabetic patients, but it has been little studied. Other factors contribute to the deterioration of QoL in diabetic patients. First, PAD patients with more metabolic syndrome components have a worse quality of life [34]. Second, a recent longitudinal study has shown that the QoL of diabetic patients declined over 5 years, due, among other reasons, to the development of complications [35]. Thus, the development of nephropathy [30], retinopathy and neuropathy are predictors of lower QoL [31].

**Figure 2:**
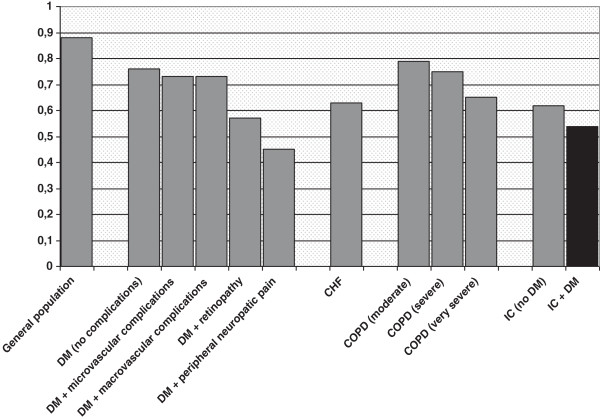
**QoL measured by EQ-5D in several chronic diseases.** DM = Diabetes Mellitus (Janssen et al. 2001); COPD = Chronic Obstructive Pulmonary Disease (Rutten-van Mölken et al. 2006); CHF = Chronic Heart Failure (de Rivas et al. 2008); IC = Intermittent Claudication (present study).

Two systematic reviews of QoL and IC revealed that deterioration in QoL affected the physical domain more than the psychological and social domains [36,37]. Lower ABI values are associated with significantly lower physical activity [22,38]. In fact, for some authors, the objective approach most closely correlated with QoL is the walking capability by treadmill test [21,22] or WIQ [22,38]. In contrast, QoL in diabetic claudicants was worse than in non-diabetic claudicants but was comparable to patients with severe chronic disease [31,39-41] (Figure 2).

Our study had several characteristics that differentiate it from other studies with similar aims: a) the large number of patients (n = 920 claudicants), which increases the reliability of our results; b) our study covered the whole country, thereby encompassing cultural variability; and c) the scoring of several parameters. Finally, the biggest potential limitation of our study was the lack of the treadmill test. However, PAD and WIQ are well correlated.

In conclusion, patients with IC and diabetes had more risk cardiovascular factors, cardiovascular conditions, disability and a poorer haemodynamic status and QoL than claudicant patients without diabetes.

## Competing interests

The authors declare that they have no competing interests.

## Authors’ contributions

Conception and design: FSL, JRM, EC, JML. Data collection: Grupo Saned. Statistical analysis of results: Grupo Saned, FSL. Analysis and interpretation of results: FSL, JRM, EC, JML. Writing up article (text, tables and figures): FSL, JRGP. Final approval of the article: FSL, JRM, EC, JRGP, JML, ER.
